# Integrated Bioinformatics Analysis the Function of RNA Binding Proteins (RBPs) and Their Prognostic Value in Breast Cancer

**DOI:** 10.3389/fphar.2019.00140

**Published:** 2019-03-01

**Authors:** Ke Wang, Ling Li, Liang Fu, Yongqiang Yuan, Hongying Dai, Tianjin Zhu, Yuxi Zhou, Fang Yuan

**Affiliations:** ^1^Clinical Laboratory, Yongchuan People’s Hospital of Chongqing, Chongqing, China; ^2^Clinical Laboratory, Yongchuan Hospital of Chongqing Medical University, Chongqing, China; ^3^Yidu Cloud (Beijing) Technology Co., Ltd., Beijing, China

**Keywords:** breast cancer, RNA binding protein, integrated bioinformatics analysis, survival, prognosis

## Abstract

**Background and Purpose:** Breast cancer is one of the leading causes of death among women. RNA binding proteins (RBPs) play a vital role in the progression of many cancers. Functional investigation of RBPs may contribute to elucidating the mechanisms underlying tumor initiation, progression, and invasion, therefore providing novel insights into future diagnosis, treatment, and prognosis.

**Methods:** We downloaded RNA sequencing data from the cancer genome atlas (TCGA) by UCSC Xena and identified relevant RBPs through an integrated bioinformatics analysis. We then analyzed biological processes of differentially expressed genes (DEGs) by DAVID, and established their interaction networks and performed pathway analysis through the STRING database to uncover potential biological effects of these RBPs. We also explored the relationship between these RBPs and the prognosis of breast cancer patients.

**Results:** In the present study, we obtained 1092 breast tumor samples and 113 normal controls. After data analysis, we identified 90 upregulated and 115 downregulated RBPs in breast cancer. GO and KEGG pathway analysis indicated that these significantly changed genes were mainly involved in RNA processing, splicing, localization and RNA silencing, DNA transposition regulation and methylation, alkylation, mitochondrial gene expression, and transcription regulation. In addition, some RBPs were related to histone H3K27 methylation, estrogen response, inflammatory mediators, and translation regulation. Our study also identified five RBPs associated with breast cancer prognosis. Survival analysis found that overexpression of DCAF13, EZR, and MRPL13 showed worse survival, but overexpression of APOBEC3C and EIF4E3 showed better survival.

**Conclusion:** In conclusion, we identified key RBPs of breast cancer through comprehensive bioinformatics analysis. These RBPs were involved in a variety of biological and molecular pathways in breast cancer. Furthermore, we identified five RBPs as a potential prognostic biomarker of breast cancer. Our study provided novel insights to understand breast cancer at a molecular level.

## Introduction

Breast cancer is the most commonly diagnosed cancer and a main cause of cancer death among women. In 2018, there was an estimated 2.1 million newly diagnosed female breast cancer cases worldwide, accounting for about 25% of cancer cases among women (Bray et al., 2018). In recent years, with great progress in medical technology, the diagnosis incidence of breast cancer has increased year by year, and the age of onset or diagnosis has consequently become younger. Breast cancer is aggressive and has a high recurrence rate. Currently, the diagnosis of breast cancer mainly relies on pathological assessments, imaging tests, and tumor markers (McDonald et al., 2016), which creates difficulty for meeting clinical requirements. In order to reduce the recurrence rate and mortality of breast cancer patients, and to improve their quality of life, it is vital to increase ability in surveillance, early detection and diagnosis. Over the years there has been an increase of molecular research on early diagnosis, drug resistance and prognosis, and it is therefore valuable to find new molecular markers on the occurrence, progression, and prognosis, to further expand this research.

RNA-binding proteins (RBPs) are abundant and ubiquitously expressed in cells. They play a central and conserved role in gene regulation (Gerstberger et al., 2014b), and act as important participants and coordinators to maintain genome integrity (Nishida et al., 2017). RBPs have extensive capabilities including regulating stability, maturation, posttranscriptional regulation of mRNA stability, splicing, editing and translation, mRNA localization and polyadenylation, which ultimately impacts the expression of every gene in the cell (Campos-Melo et al., 2014; Gerstberger et al., 2014a). Although it is known that post-transcription contributes to tumor initiation and progression, the role of RBPs in cancer remain relatively unexplored (Wurth and Gebauer, 2015).

There is a large number of Human RBPs, but very few have been studied in depth, such as AGO2, Nova, PTB, HuR, AUF1, TTP, CUGBP2 which are known for their role in many regulation processes, including interacting with non-coding RNAs (Iadevaia and Gerber, 2015), controlling intracellular localization of non-coding RNAs (Glisovic et al., 2008), methylation (Harvey et al., 2017), forming the RNA induce silencing complex (Connerty et al., 2015), and alternative splicing (Paronetto et al., 2010). RBPs participate in comprehensive biological processes, such as reproductive development, tumorigenesis and apoptosis, and is therefore closely related to many human diseases. A systematic functional study of RBPs will be helpful to understand the function and mechanism of non-coding RNA, but will also have a significant applied value in studying the pathogenesis of diseases and in the screening of innovative drug targets.

Currently, genes and signaling pathways that participate in breast cancer tumorigenesis and progression remain to be further investigated. Exploring new genes and pathways associated with breast cancer may help to identify potential molecular mechanisms, diagnostic markers and therapeutic targets (Wang et al., 2018). High-throughput genomic analysis techniques can be applied to screening for differentially expressed genes (DEGs) and to understand the relevant pathways and protein interaction networks (Vogelstein et al., 2013). In this study, we downloaded breast cancer data from the cancer genome atlas (TCGA), and selected differential expressed RBPs to perform gene ontology (GO), KEGG pathways and an interaction network and survival analysis. The study identified a number of RBPs involved in breast cancer. Some of which might be used as potential prognostic biomarkers in the future.

## Results

### Identification of Differently Expressed RBPs (DEGs)

**FIGURE 1:**
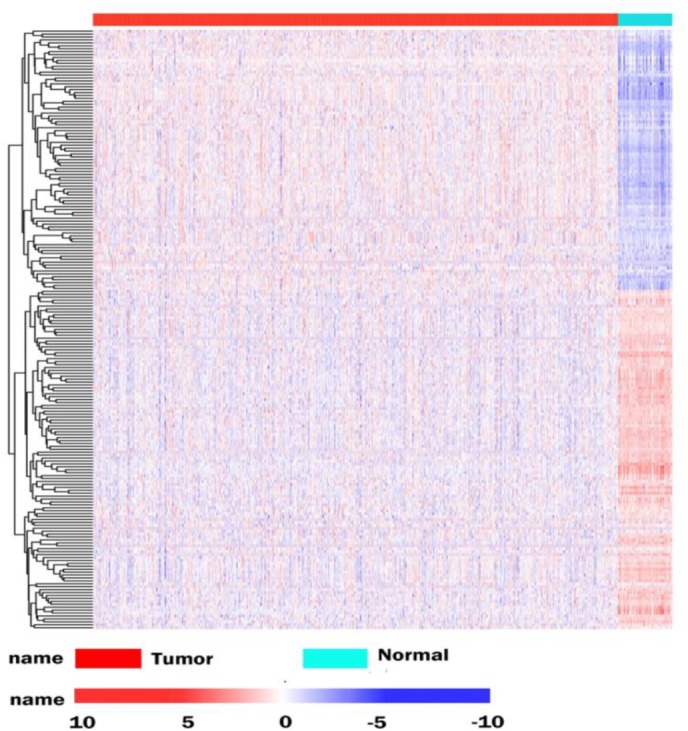
Differentially expressed RBPs in BRCA cancer. Unsupervised clustering analysis was performed using the pheatmap function, using complete and Euclidean as metrics in R, based on log2-transformed FPKM values. The columns are samples and the rows are RBPs. The blue represents down-regulation, while red represents up-regulation.

The database analysis contained 1092 breast tumor samples and 113 no-tumor control samples. We conducted a deep analysis of 1912 RBPs and a total of 205 RBPs were identified, including 90 upregulated and 115 downregulated RBPs (Supplementary Table S1). We also constructed an expression heat map for all DEGs (Figure 1).

### Functional and Pathway Enrichment Analysis of DEGs

To determine the function and mechanisms of these RBPs, all DEGs were divided into two groups (upregulated group and down-regulated group), and submitted to the David database for GO analysis. We then conducted a KEGG pathway analysis for all DEGs. We found that upregulated DEGs were significantly enriched in RNA processing, RNA binding, mRNA binding, and located in the non-membrane-bounded organelle, intracellular non-membrane-bounded organelle, ribonucleoprotein complex, intracellular organelle lumen, organelle lumen, membrane-enclosed lumen, nuclear lumen, and the nucleolus (Table 1). The GO result of down-regulated DEGs were significantly enriched in the RNA processing, posttranscriptional regulation of gene expression, mRNA metabolic process, mRNA processing, regulation of translation and the RNA binding, and these genes mainly consisted of the chromatoid body, P granule, germ plasm, pole plasm, intracellular non-membrane-bounded organelle and the non-membrane-bounded organelle (Table 1). According to the KEGG pathway enrichment analysis, all DEGs mainly participated in Dorso-ventral axis formation, fatty acid elongation in mitochondria and pathogenic *Escherichia coli* infection (Table 2).

**Table 1:** GO enrichment analysis results of differentially up-regulated genes and down-regulated genes (DEGs).

	Term	*P* value	FDR
Up-regulated genes (DEGs)	RNA processing	3.23E-06	0.00478286
	Non-membrane-bounded organelle	4.53E-13	5.56E-10
	Intracellular non-membrane-bounded organelle	4.53E-13	5.56E-10
	Ribonucleoprotein complex	5.00E-11	6.15E-08
	Intracellular organelle lumen	1.81E-09	2.22E-06
	Organelle lumen	3.09E-09	3.80E-06
	Membrane-enclosed lumen	4.90E-09	6.02E-06
	Nuclear lumen	1.32E-06	0.00162684
	Nucleolus	1.59E-06	0.00194848
	RNA binding	5.78E-16	6.77E-13
	mRNA binding	2.37E-05	0.02904478
Down-regulated genes (DEGs)	RNA processing	1.41E-10	2.21E-07
	Posttranscriptional regulation of gene expression	2.29E-09	3.58E-06
	mRNA metabolic process	1.81E-08	2.82E-05
	mRNA processing	2.40E-07	3.74E-04
	Regulation of translation	1.05E-06	0.00164623
	Chromatoid body	2.05E-06	0.00250038
	P granule	3.57E-06	0.00436073
	Germ plasm	3.57E-06	0.00436073
	Pole plasm	3.57E-06	0.00436073
	Intracellular non-membrane-bounded organelle	1.94E-05	0.02370618
	Non-membrane-bounded organelle	1.94E-05	0.02370618
	RNA binding	1.46E-23	1.83E-20

**Table 2:** The KEGG pathway analysis of all DEGs.

Term	*P* value
Dorso-ventral axis formation	4.18E-03
Fatty acid elongation in mitochondria	3.11E-02
Pathogenic *Escherichia coli* infection	2.06E-02

### Protein-Protein Interaction Network Building and Interrelation Analysis Between Pathways

**FIGURE 2:**
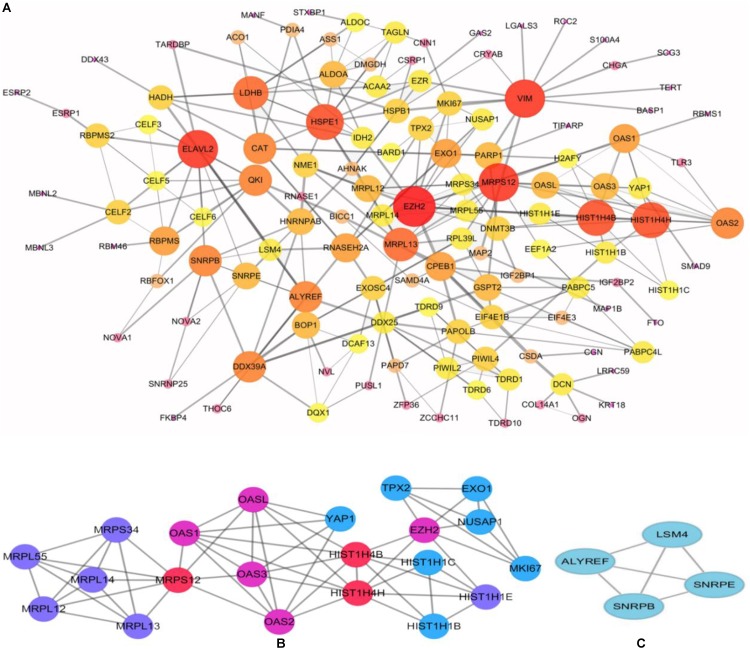
Construction of protein-protein interaction (PPI) network. **(A)** Modules inferred from protein-protein interaction network. Elected important modules of target gene (**B**,**C)** MCODE score >4, nodes >4.

**FIGURE 3:**
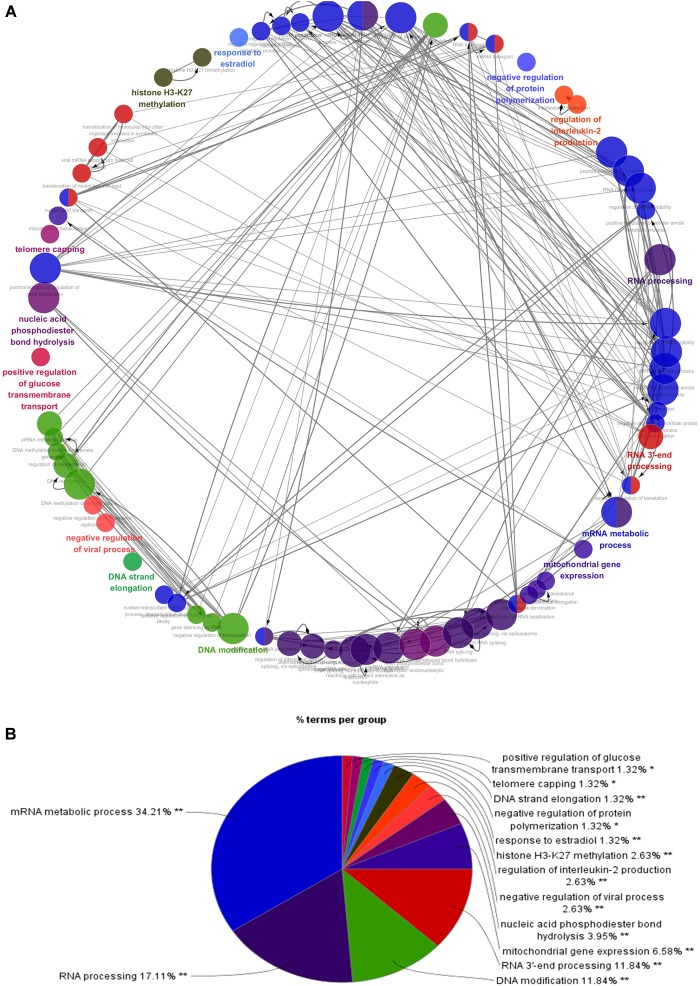
Interrelation analysis between pathways (biological process). **(A)** Interrelation between biological process pathways. **(B)** The proportion of each pathway.

**FIGURE 4:**
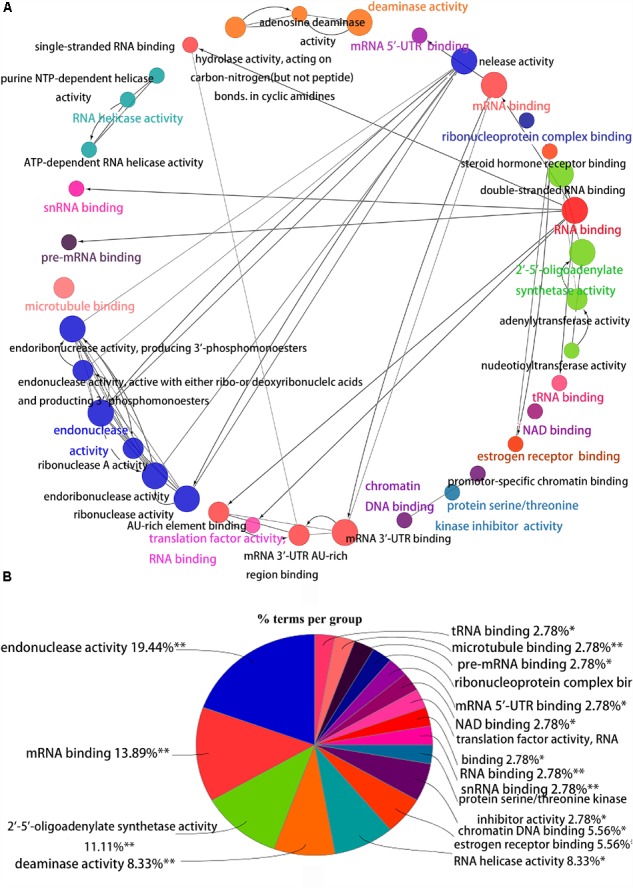
Interrelation analysis between pathways molecular functions. **(A)** Interrelation between molecular functions pathways. **(B)** The proportion of each pathway.

To better understand the role of these differentially expressed RBPS in breast cancer development, we constructed co-expression networks. All DEGs were submitted to STRING 10.5, we obtained 294 PPI nodes, 174 edges, and a *p*-value of PPI concentration <1.00–16, while also including the result of the GO and KEGG pathway. In the biological process, there was mainly enrichment in the regulation of transcription, translation level and epigenetics, and it also played an important role in histone modification, mitochondrial gene expression, cell metabolism, production of inflammatory mediators and estrogen response. The cellular components are significantly located in the ribosome, mitochondria, chromosomes, and the telomeres, etc. Molecular functions showed that they can bind to a variety of RNA and specific regions, and were closely related to regulated enzymes activity, including various metabolic and gene expressions, modification and regulation of enzymes, and also bound to steroid hormones and estrogen receptors. For KEGG pathway analysis, it was mainly enriched in Glycolysis/Gluconeogenesis, mRNA surveillance pathway, RNA degradation and pathogenic *E. coli* infection. Then, we constructed the PPI network of these DEGs using Cytoscape (Figure 2A). Two topological features, degree and betweenness, were calculated to identify candidate hub nodes. The higher the two quantitative values of a gene, the greater the importance within the network (Liu et al., 2018b). The co-expression network revealed that ELAVL2, VIM, MRPS12, HSPE1, EZH2, HIST1H4B, and MRPL13 played a vital role in the progression of breast cancer, and we further selected important modules of target genes through MCODE (Figure 2B,C). Finally, we used the ClueGO to externalize all biological processes (Figure 3A,B) and the interaction modes of molecular functions (Figure 4A,B).

### Survival Analysis

**FIGURE 5:**
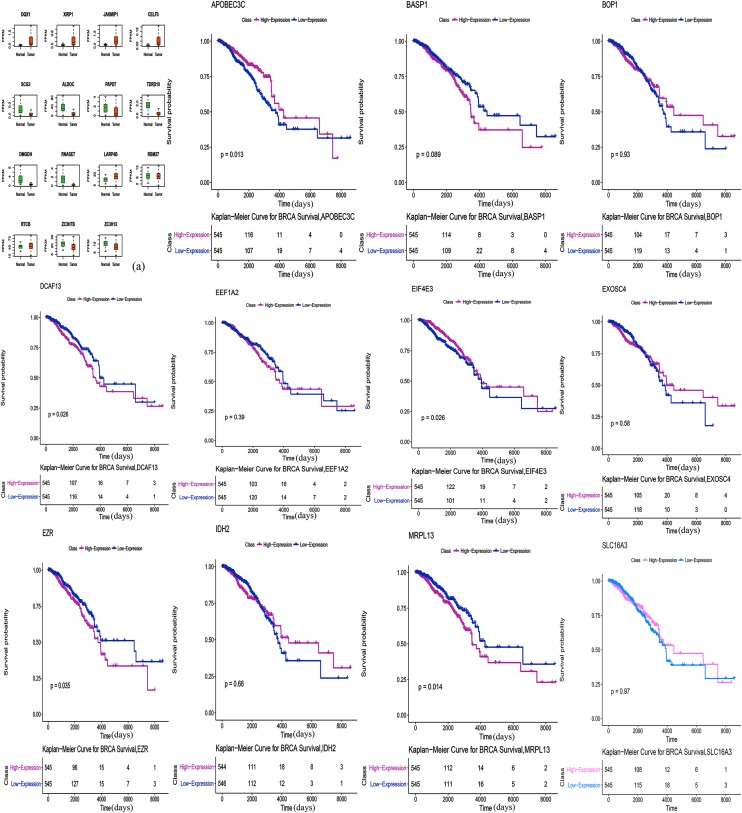
Survival Kaplan-Meier Estimate (EZR *p* = 0.035, DCAF13 *p* = 0.026, MRPL13 *p* = 0.014, APOBEC3C *p* = 0.013, and EIF4E3 *p* = 0.026). Expression of selected target genes in tumor and normal tissues was significantly different.

The correlation between RBP expression and overall survival was assessed using both the Cox regression analysis and the Kaplan-Meier estimation method. Then, survival correlation *P* < 0.05 and key RBPs were selected to analyze their correlation with survival prognosis. After that we used both the Kaplan-Meier estimates and the log-rank test to assess the significant differences of the two-group survival curves. As shown in the Figure 5, the expression of selected target genes in tumor and normal tissues was significantly different. In addition, patients with highly expressed RBPs of EZR, DCAF13, and MRPL13 showed lower survival, but patients with highly expressed RBPs of APOBEC3C, EIF4E3 showed better survival (Figure 5). Therefore these genes could be potential biomarkers for breast cancer prognosis.

## Materials and Methods

### Data Sources Analysis

The corresponding clinical data was downloaded from the following website http://gdac.broadinstitute.org/. Combined with the RNA-seq expression data set, 1075 patients had clinical information available including age, gender and disease stage. The details have been listed in Supplementary Table S1. We downloaded the RBPs expression data (TOIL RSEM expected count and FPKM) processed by the Toil pipeline (Vivian et al., 2017) based on RNA sequencing (RNA-Seq) for TCGA Pan-Cancer cohort from the website https://xena.ucsc.edu/. The data included the 60498 genes annotated by GENCODE version 23. We then used custom Perl script to extract the data from BRCA cancer, for subsequent analysis. We applied the Voom function (Law et al., 2014) in the Limma package, to estimate DEGs between tumor and normal tissues for BRCA cancer. Those with a fold change ≥ 1 and FDR < 0.05 were considered to have statistical significance. We further identified significantly dysregulated RBPs based on our RBP catalog. Unsupervised clustering of differentially expressed RBPs was performed based on log2-transformed FPKM values using the “pheatmap” package in R.

### GO Functional and Pathway Enrichment Analysis

In order to comprehensively analyze the biological functions of these RBPS, we used the GO and kyoto encyclopedia of genes and genomes (KEGG) analysis by The database for annotation, visualization and integrated discovery (DAVID) version 6.7. The GO Term analysis included the biological process, cellular component and the molecular function. Both *P* < 0.05 and FDR < 0.05 were considered statistically significant.

### Protein Interaction Network (PPI) and Pathways Interaction Analysis Building

SRTING version 10.5 was used to evaluate the protein interaction information of all DEGs, and their biological functions were also obtained. Then, the interaction network of these proteins was visualized by Cytoscape3.6.0, and important modules both MCODE score and node number > 4 were selected by the MCODE plug in to Cytoscape version 3.6.0. Furthermore, the pathway enrichment of *P* < 0.05 was analyzed by the ClueGO plug to Cytoscape version 3.6.0.

### Statistical Analysis

The correlation of RBP expression and overall survival was assessed using both the Cox regression analysis and the Kaplan-Meier estimation method, based on the “survival” package in R. For the Cox regression analysis, the RBP was evaluated as a continuous variable with age and gender as additional covariables. For the Kaplan-Meier estimates, we defined the high-expression group and low-expression group using the median RBP expression value as a cut-off point. A significant difference of two-group survival curves was assessed by a log-rank test. *P* < 0.05 was considered as statistically significant.

## Discussion

Currently, cancer causes more death than coronary heart diseases or stroke does (Lin et al., 2017a). In recent years, although molecular targeted therapy has improved treatment effect, breast cancer is still the primary cause of death among women. During clinical practice, biomarkers that indicate the grade malignancy, metastasis and the prognosis of breast cancer are needed. Microarray and high-throughput sequencing technologies provide effective tools for deciphering key genetic or epigenetic changes in the occurrence of cancer, as well as promising biomarkers for cancer diagnosis, treatment, and prognosis (Kulasingam and Diamandis, 2008; Cancer Genome Atlas Research and Network, 2014). Our study integrated TCGA RNA sequencing data, and identified DEGs between tumor and normal tissue. We analyzed relevant biological pathways, constructed protein interaction networks and performed survival analyses to explore biological functions and clinical application of these RBPs.

The biological functions of these DEGs were obtained using the GO and KEGG pathway analysis. Firstly, the enrichment of cell components is mainly located in the ribosome, exonuclease, endonuclease, spliceosome and the ribonuclease, which are important sites protecting the transmission of biological information. The ribosome is a key organelle that performs protein synthesis. The mutation of the ribosomal protein regulates the translation and activity of p53, finally resulting in diseases, and cancers (Goudarzi and Lindstrom, 2016). A number of RBPs exist in exonuclease, endonuclease and sites with DNA damage, which may participate in DNA damage repair (Goudarzi and Lindstrom, 2016). In addition, RBPs are widely present in spliceosome. Expression of eukaryotic genes is often accompanied by the RNA splicing process, especially in the alternative splicing of RNA, which could produce tissue and development specific mRNA. For example, Sam68 can result in drug resistance and poor prognosis by regulating the expression ratio of cyclinD1, an alternative splicing in breast cancer (Paronetto et al., 2010). Some RBPs also expressed in the telomere and telomerase and regulate their activity. Telomere play an important role in regulating cell growth and division. Some studies have found that telomerase activity was suppressed in normal tissues but was reactivated in tumors. Telomerase is overexpressed in 80–95% of cancers and is likely to participate in cell malignant transformation (Ruden and Puri, 2013). During the analysis of cellular components, we also found the occurrence of RBPs in the exosome, which could cause tumor invasion and metastasis, immune escape and therapeutic resistance. For example, SYNCRIP, as a component of the miRNA sorting mechanism, in hepatocyte exosomes, can directly bind to specific miRNA rich in exosomes, and regulates miRNA localization (Santangelo et al., 2016).

Secondly, in terms of molecular function, RPBs can bind to various RNAs such as pre-mRNA, Sn RNA, tRNA, mRNA and regulates the activity of various enzymes, such as hydrolytic enzyme, purine metabolic enzyme, and enzymes involved in DNA synthesis, repair, and RNA metabolism. Furthermore, some RBPs also bind to estrogen and steroid hormone receptors. For example, MSI2 is highly expressed in ER(+) breast cancer, and its expression is significantly correlated with ESR1 expression, which affects the growth of breast cancer cells, by changing the function of ESR1 (Kang et al., 2017).

Next, for the biological process, the function enrichment of differential RBPs mainly occurred in RNA processing, splicing, localization, transport, hydrolysis, and RNA silencing. It participates in transposition regulation, methylation, and alkylation of DNA. Some RBPs were also related to histone H3K27 methylation, inflammatory mediators, and translation regulation. Our findings are consistent with the consensus that multiple genes, multiple molecules, and multiple pathways are involved in breast cancer. Although the relationship with breast cancer remains unclear, some RBPs have been reported in other cancers. HuR can promote the growth of colorectal cancer cell by regulating mRNA expression (Lopez de Silanes et al., 2003). CRD-BP can regulate many mRNAs with coding for cancer-related genes, including Gli1, PTEN, PTEN, ptlcp1, MAPK4, MDR1, IGF2, H19, c-myc, etc. (Fakhraldeen et al., 2015). HNRNPA2B1 controls the replacement splicing for the pre-mRNA of cancer-related genes, and which is up-regulated in diverse cancers (Stockley et al., 2014). HuR can bind with DNMT3b and maintain its stability, thus affecting abnormal DNA methylation (Lopez de Silanes et al., 2009). Numerous studies reported that a change of mitochondrial function plays a key role in all kinds of cancers (Tao et al., 2015; Lin et al., 2017b; Zhang J.Y. et al., 2017; Zhang J. et al., 2017), and RBPs involved in the expression and transcription regulation of mitochondrial genes, such as LRPPRC, GRSF1, SLIRP, and other RBPs can interact with mt-RNA to affect the expression and metabolism of mitochondrial transcripts (Dong et al., 2017). The incidence of breast cancer and female estrogen levels are closely related. Some RBPs can respond with estrogen, for example, through Nova1, 17-b estradiol can regulate the replacement splicing of estrogen receptor b in the brain of aging female mice (Shults et al., 2018). Then, the results of the KEGG pathway analysis indicated that these RBPs may affect the occurrence and development of breast cancer through glycolysis, glycosylation, mRNA monitoring pathways, and RNA degradation regulation. RPBs have various basic biological functions, especially the function of RNA which has been studied widely. Other RBPs functions should therefore be studies further.

By constructing a protein network for DEGs, we found that breast cancer is associated with immune response, splicing, transcript regulation, and intercellular signaling transduction. HSPE1 is a member of the heat shock protein family (Hsp10) E, which usually acts as a chaperone to assist protein folding in the mitochondria, which is highly expressed in various cancers, such as lung cancer, pancreatic cancer and bladder cancer. Some studies reported that it may protect cancer cells from apoptosis, and facilitate the immune escape of cancer, by down-regulating the immune response (Rappa et al., 2016; Liu et al., 2018a). ELAVL2 is a neurospecific RNA binding protein, which is involved in splicing and transcript trafficking to regulate protein localization (Berto et al., 2016). Elevated methylation of ELAVL2 was shown in high Gleason scores of prostate tumors (Wu et al., 2016). VIM is expressed in a variety of cell types and is responsible for maintaining cell shape, cytoplasmic integrity, and stabilizing cytoskeletal. It is also involved in immune responses, attachment, migration, and cell signaling in tissues. Previous studies have shown that vimentin regulated Ras, Slug and TGF glows in cancer cells, which is necessary for EMT induction. It is also highly expressed in various tumors such as lung cancer, breast cancer and gastric cancer, and is closely related to invasion, metastasis and the poor prognosis of tumors (Satelli and Li, 2011; Virtakoivu et al., 2015). High expressions of EZH2 is associated with malignancy and hyper-invasiveness in a variety of cancers. EZH2 can activate NF-κB targets and NOTCH1 in breast cancer cells, which has also been implicated in the transcriptional activation of gene expression in breast cancer. Research has shown that it induces the expression of genes that are regulated by the estrogen receptor (ER) and Wnt signaling transcription factors, by physically bridging between the ER and components of Wnt signaling (Kim and Roberts, 2016).

These RBPS may lead to breast cancer by regulating mitochondrial translation, splicing of pre-mRNA, activation of RNase L, and histone modifications through two modules selected from the PPI network. It has been reported that the upregulation of mitochondrial translation may meet the energy needs of cancer cells in human tumors, but the mechanism of its tumorigenesis remains unclear. There are many studies targeting the inhibition of mitochondrial translation in various cell types, to obliterate cancer stem cells. Currently, suppressing mitochondrial translation is considered a valuable therapeutic target (Kim et al., 2017). RNase L was activated through the synthesis of 2′, 5′ -oligoadenylic acid by OAS (OAS1, OAS2, and OAS3). It was found that the activated OAS-RNase L system can degrade virus and cell RNA, promote cell apoptosis, and inhibit protein synthesis (Bhosle et al., 2016). In addition, single nucleotide polymorphisms of OAS are associated with cancer, such as OAS1 SNP rs2660 AA (Mandal et al., 2011). However, no studies reported the exact role of OAS in breast cancer. As we know, epigenetic change is involved in the initiation and progression of cancer, which includes histone modifications and DNA methylation. Studies have shown that the regulation of histone is gene specific, but their function is diversified. Histone cluster 1 can interact with some regulatory factors, such as inhibiting p53-dependent chromatin transcription, and maintaining or establishing specific DNA methylation patterns (Perez-Magan et al., 2010). It has been demonstrated that the function of protecting DNA with histone may be an independent prognostic factor for better survival of cervical cancer patients (Li et al., 2017). Furthermore, splicing affects the expression of most genes, and eventually influences the levels of proteins. In the module, SNRPE, SNRPB, and ALYREF participate in the splicing of pre-mRNA. Knockdown SNRPE significantly reduces the expressed level of mTOR mRNA and protein, and is accompanied by the imbalance of the mTOR pathway, which activates abnormal mTOR signaling and which can result in the growth and metastasis of tumor cells (Quidville et al., 2013).

Finally, we performed a survival analysis and found five genes that are associated with survival in breast cancer patients. The overexpression of DCAF13, EZR, and MRPL13 in patients were associated with lower survival, which reveals that these genes might be associated with tumor invasion, progression and poor prognosis. Whereas, overexpression of APOBEC3C and EIF4E3 in patients were associated with better survival, suggesting their potential role as tumor suppressor genes. DCAF13 is amplified in all kinds of cancers. Studies have shown that overexpression of DCAF13 in hepatocellular carcinoma is significantly correlated with low survival and it may be involved in the regulation of cell cycle (Cao et al., 2017). It also reported that the E3 ligase formed by DCAF13, CUL4B and DDB1, could induce ubiquitination of tumor suppressor PTEN *in vivo* and *in vitro* (Chen et al., 2018). Mutated or inactivated PTEN was helpful to infiltrate and spread cancer cells. As a member of the ERM protein family, Ezrin has been linked to molecules that control the phosphatidylinositol-3-kinase, AKT, Erk1/2 MAPK and Rho pathways, which are functionally involved in regulating cell survival, proliferation and migration, and it is an indicator of poor prognosis of multiple cancers (Hunter, 2004). It has been shown that overexpressed EZR in a nude mice phantom of pancreatic cancer, can increase the number of metastasis and is closely related to the progression of malignant cancer (Meng et al., 2010). MRPL13 is a mitochondrial ribosomal protein. Loss of MRPL13 can lead to the loss of mitochondrial DNA, and eventually lead to the loss of the ability of mitochondrial coding proteins (Gruschke et al., 2010). In a study, reduced MRPL13 expression in hepatocellular carcinoma was a key factor in the regulation of mitochondrial ribosome and subsequent OXPHOS deficiency, which regulates the aggressive activity of liver cancer cells (Lee et al., 2017). APOBEC can mediate c-to-t mutagenesis in various cancers, while the APOBEC3 gene family is overexpressed in breast cancer and other cancer cells and tissues. Some studies suggest that it is regulated by estrogen in breast cancer (Long et al., 2013). At present, there are few studies about APOBEC3C in breast cancer, and some studies have found that it should play a positive role in the invasiveness and prognosis of hepatocellular carcinoma (Zhang et al., 2015). EIF4E3 belongs to the EIF4E family of translational initiation factors that interact with the 5-prime cap structure of mRNA. A study demonstrated that EIF4E3 relies on cap-binding activity to act as a tumor suppressor and compete with the growth-promoting functions of EIF4E. In fact, reduced EIF4E3 levels in high-expressed EIF4E cancers suggests that EIF4E3 underlies a clinically relevant inhibitory mechanism that is lost in some malignancies (Osborne et al., 2013). Other studies also found that EIF4E3 can impede oncogenic transformation (Volpon et al., 2013).

Over all, we identified key genes and related pathways through bioinformatics analysis of differential expressions of RBPs in breast cancer. These RBPs may be involved in the occurrence, development, invasion and metastasis of breast cancer. The survival analysis suggested that DCAF13, EZR, MRPL13, APOBEC3C, and EIF4E3 might have a prognostic value for breast cancer. Future *in vitro* and *in vivo* studies are needed to verify the functions of these genes.

## Data Availability

Publicly available datasets were analyzed in this study. This data can be found here: https://xena.ucsc.edu/.

## Author Contributions

LL and KW conceived and designed the experiments. LF, YY, TZ, and HD analyzed the data. LL,YZ, and FY wrote the manuscript. All authors reviewed and approved the final manuscript.

## Conflict of Interest Statement

YZ and FY were employed by company Yidu Cloud Technology Co. The remaining authors declare that the research was conducted in the absence of any commercial or financial relationships that could be construed as a potential conflict of interest.
