# Supplementary material for: Integrated Bioinformatics Analysis the Function of RNA Binding Proteins (RBPs) and Their Prognostic Value in Breast Cancer
Source: Front Pharmacol. 2019 Mar 1;10:140. doi: 10.3389/fphar.2019.00140 (PMC6405693; doi:10.3389/fphar.2019.00140)
Supplement: Supplementary file 1 [file Table_1.docx]

Table 1 Compared with non-tumorous samples, 205 DEGs were identified from the datasets analyzed, of which 90 genes were up-regulated genes and 115 genes were down-regulated genes in breast cancer tissues.

| DEGs | RBPs |
| --- | --- |
| Upregulated | EXO1, TPX2, MKI67, RDM1, DQX1, NUSAP1, EEF1A2, HIST1H4H  ESRP1, XIRP1, EZH2, SLC16A3, JAKMIP1, KRT18, CELF3, TERT, KNOP1  IGF2BP1, MEX3A, HIST1H1C, HIST1H1E,HIST1H1B,RNASEH2A,OASL, CGN, NME1, MKRN3, ELAVL2, ESRP2, MAZ, FKBP4, HMGA1, MRPS34  DDX39A, EXOSC4, EZR, LSM4, RPUSD1, SNRNP25, BOP1,HSPB1,IDH2  NUDT16L1, OAS2, MRPL14, OAS3, TSEN54, SNRPB, RNASE2, MRPL55  NVL, EIF4E1B, DNMT3B, COA6, DNAJC1,HDGF, MRPS12, PARP1, GP2  LRRC59, PUSL1, INTS7, STRBP, ALYREF, C1orf35, BARD1, CD3EAP  PDIA4, ZNF239, RPL39L, FLYWCH2, BASP1, MRPL12, ARPC1B HNRNPAB, OAS1, SNRPE, DCAF13, HSPE1, THOC6, ADAT3, MRPL13  RCC2, MANF, CCDC137, MTCL1, H2AFY, PTRH1, HIST1H4B, ALDOA |
| Downregulated | ZCCHC11, SNTB2, NYNRIN, PABPC4L, CTIF, RBFOX1, DZIP1, LGALS3  S100A4, RBM20, FTO , RBMS1 , CELF6 , APOBEC3C , ACAA2 , EIF4E3  HADH, HABP4, PAPOLB, SRSF12, CELF5, C15orf52, APOBEC2, RBPMS  DZIP1L, RBMS2, ASS1, TDRD9, TIPARP, YAP1, RNASE1, CSRP1, YBX3  QKI, PPARGC1B, TARDBP, ADARB2, TLR3, FBXO17, TDRD6, MBNL2 BICC1, STXBP1, MBNL3, ZCCHC5, DDX26B, ARHGEF28, ZNF106, VIM PABPC5, TDRD1, ZFP36L2, MAP2, MAP3K20, RBPMS2, PIWIL4, LDHB RNASE13, NOVA2, RNASE4, PCBP3, MAP1B, TAGLN, CHGA, ADAD2 ACO1, LRP1, AFF2, ZCCHC24, GSPT2, SORBS2, RBM46, SYNE1, ADD3 AHNAK, NPM2, SMAD9, ZC3H12B, DDX25, SAMD4A, ZC3H12C, CAT DDX43, LARP6, SPTBN1, ENDOU, IGF2BP2, PTRF, DCD, PIWIL2, DCN SCG3, CELF2, CPEB1, GAS2, LAMA2, ZFP36, COL14A1, ALDOC, DST PAPD7, RBMS3, TDRD10, PPARGC1A ,NOVA1, NDRG2, NR0B1, OGN DMGDH, TNS1, SORBS1, CNN1, RNASE7, CRYAB, C4BPA |
